# Dynamic spatio‐temporal contribution of single β5t+ cortical epithelial precursors to the thymus medulla

**DOI:** 10.1002/eji.201545995

**Published:** 2016-01-18

**Authors:** Carlos E. Mayer, Saulius Žuklys, Saule Zhanybekova, Izumi Ohigashi, Hong‐Ying Teh, Stephen N. Sansom, Noriko Shikama‐Dorn, Katrin Hafen, Iain C. Macaulay, Mary E. Deadman, Chris P. Ponting, Yousuke Takahama, Georg A. Holländer

**Affiliations:** ^1^ Department of BiomedicineUniversity of BaselBaselSwitzerland; ^2^ Division of Experimental Immunology, Institute for Genome ResearchUniversity of TokushimaJapan; ^3^ The Kennedy Institute of RheumatologyUniversity of OxfordOxfordUK; ^4^ Wellcome Trust Sanger Institute‐EBI Single Cell Genomics CentreWellcome Trust Sanger InstituteHinxtonCambridgeUK; ^5^ MRC Functional Genomics Unit, Department of Physiology, Anatomy and GeneticsUniversity of OxfordOxfordUK; ^6^ Department of Paediatrics and the Weatherall Institute of Molecular MedicineUniversity of OxfordOxfordUK

**Keywords:** β5t, Development, Epithelial cell, Medulla, Thymic progenitor cell

## Abstract

Intrathymic T‐cell development is critically dependent on cortical and medullary thymic epithelial cells (TECs). Both epithelial subsets originate during early thymus organogenesis from progenitor cells that express the thymoproteasome subunit β5t, a typical feature of cortical TECs. Using in vivo lineage fate mapping, we demonstrate in mice that β5t^+^ TEC progenitors give rise to the medullary TEC compartment early in life but significantly limit their contribution once the medulla has completely formed. Lineage‐tracing studies at single cell resolution demonstrate for young mice that the postnatal medulla is expanded from individual β5t^+^ cortical progenitors located at the cortico‐medullary junction. These results therefore not only define a developmental window during which the expansion of medulla is efficiently enabled by progenitors resident in the thymic cortex, but also reveal the spatio‐temporal dynamics that control the growth of the thymic medulla.

## Introduction

The thymus provides the physiological microenvironment for the development of T lymphocytes and is therefore crucial for the immune system's ability to distinguish between vital self and injurious nonself. Essential for this competence are thymic epithelial cells (TECs), which classify into separate cortical (c) and medullary (m) lineages with specific molecular, structural, and functional characteristics 1. cTECs attract blood‐borne precursor cells, commit them to a T‐cell fate and foster their differentiation to a developmental stage at which individual immature T cells (designated thymocytes) express the TCR and can be positively selected dependent on their TCR affinity for the peptide/MHC complexes 2. In contrast, mTECs—in collaboration with dendritic and other hematopoietic cells situated in the thymic medulla—mediate both the negative selection of thymocytes that recognize self‐antigens with high affinity and the generation of regulatory T cells 3, 4, 5, 6. The instruction of a functional yet self‐tolerant T‐cell repertoire by both cTECs and mTECs depends on their collective ability to promiscuously express transcripts encoding almost all ubiquitously and tissue‐restricted proteins 7.

Both TEC lineages are derived in the embryo from a common epithelial stem/progenitor population 8, 9, 10, 11 that gives rise to cells simultaneously expressing markers characteristic for cTEC and mTEC lineages 12, 13. This finding contests a developmental model in which bipotent stem/precursor cells lacking cortical or medullary hallmarks segregate synchronously into the two different TEC lineages 14. Indeed, mTECs develop during embryogenesis from progenitors that express the cTEC prototypical marker β5t encoded by the *Psmb11* locus 15. Moreover, epithelial cells with a bipotent, self‐renewing capacity appear to persist after birth 16, 17, 18. It remains unknown whether these cells physiologically contribute to both TEC subpopulations, or whether lineage‐restricted precursors separately maintain the cTEC and mTEC compartments as in other epithelial organs where bipotent cells initially establish a multilineage epithelial structure that is later maintained by lineage‐specific, unipotent progenitors 16, 19, 20, 21, 22, 23.

Here, we report that β5t^+^ cTECs at the cortico‐medullary junction of 1‐week‐old mice serve as an efficient progenitor for the mTEC lineage. Contributions from these precursors to the medulla are multiclonal for individual medullary islands. However, once the medulla has reached its normal cellularity in the postnatal thymus, the differentiation potential of β5t^+^ precursors to the mTEC lineage is markedly restricted.

## Results

### Adult cortical and medullary thymic epithelia are derived from embryonic β5t expressing precursors

**Figure 1:**
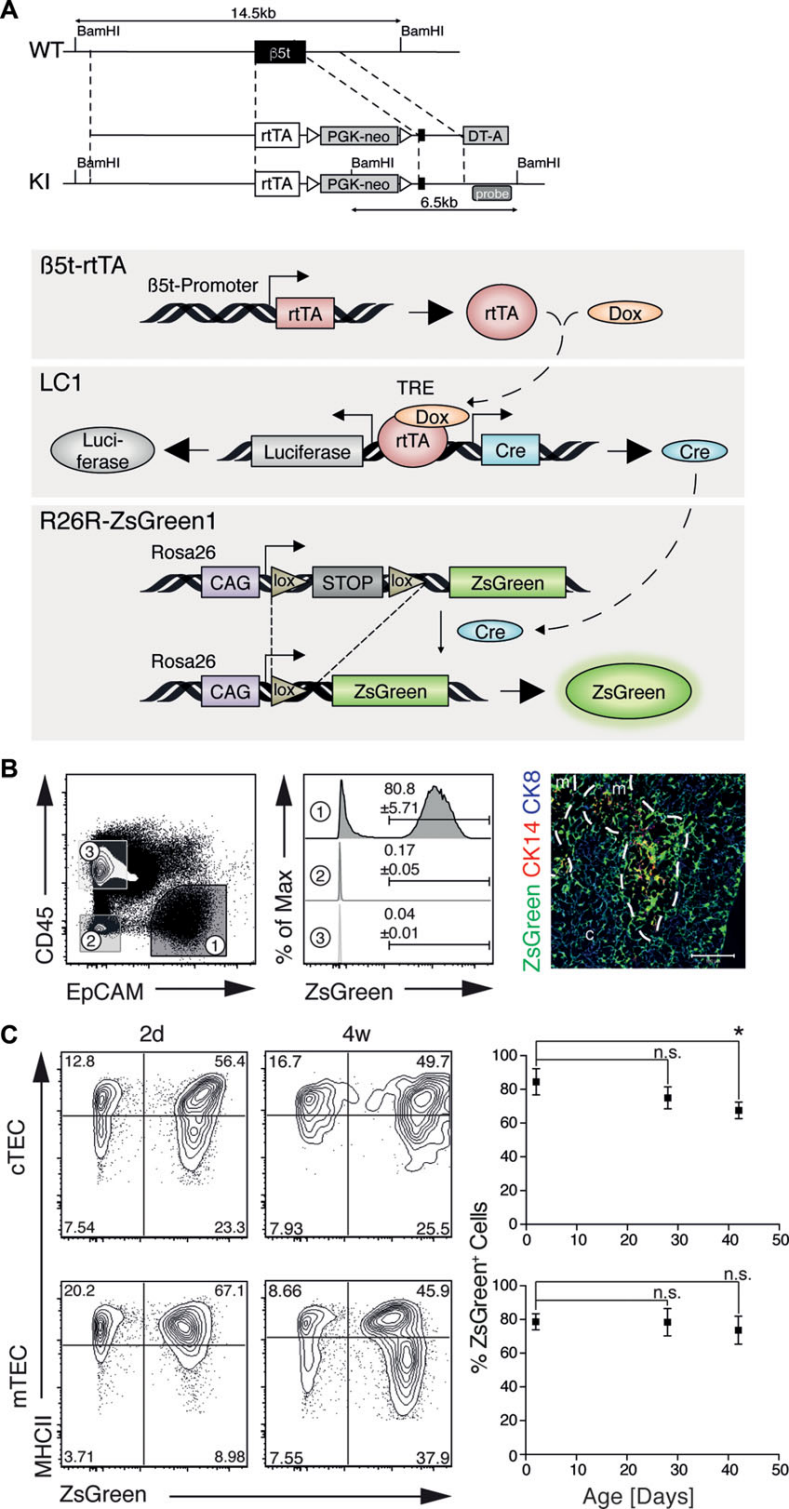
Tissue and time controlled expression of the reporter ZsGreen in thymic epithelial cells. (A) Description of the targeting strategy to achieve rtTA expression under the transcriptional control of the *β5t* locus and a cartoon depicting the design of *β5t* promoter‐driven, TEC‐specific labeling in the triple transgenic mice, designated 3xtg^β5t^. (B, C) Flow cytometric and immunofluorescent analyses of thymic tissue isolated from mice treated with Dox from embryonic day (E) 7.5 until birth. (B) The flow cytometric analyses of epithelial (EpCAM^+^CD45^−^), haematopoietic (EpCAM^−^CD45^+^), and nonepithelial stromal (EpCAM^−^CD45^−^) cells 2 days after treatment are shown. The ZsGreen expression is shown in the middle panels for each of the separate cell populations identified in the left panel. The right panel demonstrates immunofluorescence analysis of ZsGreen expression in combination with CK8 (blue) and CK14 (red). The data shown are representative of two independent experiments. (C) Flow cytometric analysis of cTECs (EpCAM^+^Ly51^+^UEA1^−^CD45^−^) and mTECs (EpCAM^+^Ly51^−^UEA1^+^CD45^−^) for expression of MHCII and ZsGreen 2 days and 4 weeks after treatment (left panels). Frequencies of ZsGreen positive cTECs and mTECs detected at indicated days after Dox treatment are displayed in the right panel (mean ± SD). The data shown are representative of 2–4 independent experiments with at least three mice per time point each. Statistical significance determined by unpaired two‐tailed Student's *t*‐test. *p < 0.05.

Given the unexpected finding that embryonic TEC precursors for both the cortical and medullary lineages express β5t 15, we set out to further identify these cells and their developmental potential throughout the life course of the mouse. For this purpose, we created a new mouse line (designated β5t‐rtTA) that expresses the reverse tetracycline transactivator (rtTA) under the transcriptional control of the β5t locus (*Psmb11*; Fig. 1A). Correctly targeted mice were crossed to animals transgenic for LC1 and the conditional ZsGreen reporter 24, 25. Treatment of these triple transgenic mice (designated 3xtg^β5t^) with doxycycline (Dox) drives the expression of Cre and consequently enables the transcription of the fluorescent protein ZsGreen in β5t‐expressing TECs and their progeny (Fig. 1A, lower panel).

3xtg^β5t^ mice were treated from embryonic day 7.5 (E7.5) until birth to ensure sufficient Dox concentration both prior to and during the formation of the thymus. This resulted in the expression of ZsGreen in the vast majority of cortical (i.e. EpCAM^+^ Ly51^+^ UEA1^−^ CD45^−^) and medullary TECs (EpCAM^+^ Ly51^−^ UEA1^+^ CD45^−^; Fig. 1B and C; Supporting Information Fig. 1) during the first 40 days of postnatal life but excluded the labeling of haematopoietic and nonepithelial stromal cells (Fig. 1B). These findings corroborated our previous finding of a collective of fetal β5t‐positive precursors from which cTECs and mTECs originate 15.

### Long‐term contribution of labeled β5t^+^ progenitors to the mTEC lineage in adult mice

**Figure 2:**
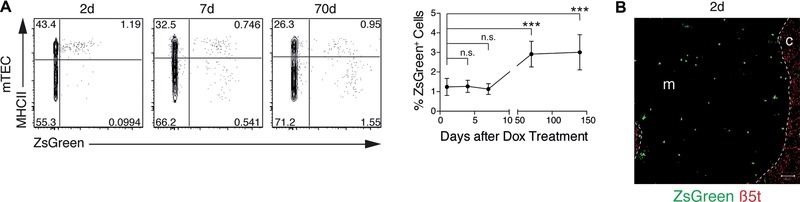
Lineage tracing of medullary thymic epithelial cells in adult mice. (A) Frequency and time course analyses of ZsGreen expression in mTECs of treated 3xtg^β5t^ mice. Five‐week‐old 3xtg^β5t^ mice were i.p. injected twice within 24 h with Dox (2 mg) and given Dox (2 mg/mL) supplemented drinking water in that time. The frequency of labeled mTECs was measured at the indicated time points. Flow cytometric analyses of mTECs (EpCAM^+^Ly51^−^UEA1^+^CD45^−^) for expression of MHCII and ZsGreen (left); relative frequency of ZsGreen+ mTECs over the course of 140 days (right, mean ± SD, *n* = 3 mice per time point, from a single experiment representative of two independent experiments per time point). Statistical significance determined by unpaired two‐tailed Student's *t*‐test. *p < 0.05, **p < 0.01, ***p < 0.001. (B) Immunofluorescent analysis of the thymic tissue at 2 days after Dox treatment of 5‐week‐old 3xtg^β5t^ mice. Tissue sections were stained with anti‐β5t antibodies (red) and analyzed for the expression of ZsGreen (green). c: cortex; m: medulla. Dashed line demarcate cortico‐medullary junction. Scale bar 50 μm.

To determine whether a comparable precursor‐progeny relationship exists for the adult thymus, we treated 5‐week‐old 3xtg^β5t^ mice with Dox for 24 h and determined ZsGreen expression in mTECs. Dox‐treatment of adult 3xtg^β5t^ mice initially resulted in ZsGreen labeling of cTECs (Supporting Information Fig. 2A and B) and mostly mature (i.e. high MHCII expressing; MHC^hi^) mTECs at very low frequency (1–2%; Fig. 2A and B). Over the course of a few days, the rate of labeled immature (MHC^lo^) cells increased and mTECs with either phenotype persisted for at least 140 days though the overall frequency of ZsGreen+ mTECs remained low (Fig. 2A and Supporting Information Fig. 2C). The low frequency of labeled cTECs in 5‐week‐old mice correlated with a reduced expression of the reverse transactivator as compared to 1‐week‐old animals (Supporting Information Fig. 3A). The labeling efficiency of adult TECs was marginally increased in mice transgenic for the tetO‐Cre transcriptional unit (β5t‐rtTA::tetO‐Cre1Jaw/J::ZsGreen, designated 3xtg^tetO‐Cre^) in lieu of the LC1 transgene (Supporting Information Fig. 3B and C) or in 3xtg^β5t^ mice treated for a longer time with Dox (Supporting Information Fig. 3D). 3xtg^tetO‐Cre^ mice were, however, considered unsuitable for our purpose owing to their spontaneous Cre‐mediated recombination (Supporting Information Fig. 3B and C). Thus, the drug‐mediated recombination had likely occurred in precursors from which immature and mature mTECs had differentiated over time. Alternatively but unlikely, a relatively small but discernable population of labeled cells may either had an extended half‐life and/or were generated by self‐duplication of existing, differentiated ZsGreen+ mTECs without any input from stem cells as described for other organ systems 26, 27, 28.

**Figure 3:**
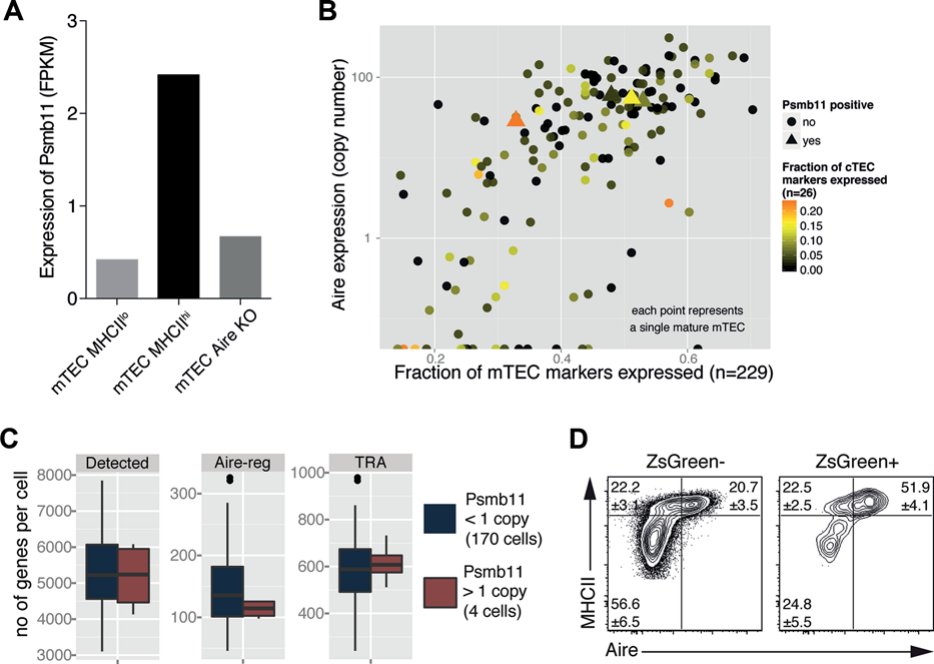
Promiscuous gene expression contributes to ZsGreen expression in medullary MHCII^hi^ thymic epithelial cells in adult 3xtg^β5t^ mice. (A) Expression of β5t (*Psmb11*) measured by RNAseq of 4‐week‐old wild‐type MHCII^hi^ mTECs, MHCII^lo^ mTECs, and MHCII^hi^ Aire‐deficient mTECs. (B) Promiscuous expression of Psmb11 in single mature mTECs. Mature mTECs positive for Psmb11 (> 1 copy, triangles) express Aire and show expression of mTEC and cTEC marker genes that is similar to that observed in Psmb11 negative cells (circles). (C) Analysis of gene expression in single Aire‐expressing mTECs. No significant difference in the number of Aire‐regulated genes or TRA was detected between Psmb11 positive and negative cells (Mann–Whitney *U* test: Detected: *p* = 0.8448; Aire‐reg: 0 = 3153; TRA: *p* = 6369). (D) Flow cytometric analysis for the expression of MHCII and Aire in ZsGreen– and ZsGreen+ mTECs of 3xtg^β5t^ mice 48 h after Dox treatment. Values indicate mean ± SD percentage of gated population (*n* = 3 mice; representative of two independent experiments).

β5t expression in mTECs is enhanced in the presence of Aire and thus part of the promiscuous gene expression programme of mature mTECs 7. Though β5t transcripts were detected in wild‐type mice in immature and mature mTECs at the population level (Fig. 3A), only a small fraction of single MHC^hi^ mTECs transcribed β5t (i.e. 4 of 174 cells with on average 6.4 mRNA copies each) (Fig. 3B). However, these β5t+ cells displayed a gene expression profile that was typical of mature mTECs as they transcribed Aire‐dependent and Aire‐independent tissue restricted antigens (TRA) comparable to the profile of other mature mTECs (Fig. 3C). Furthermore, the relative frequency of ZsGreen+ Aire+ MHC^hi^ mTECs was significantly increased in 3xtg^β5t^ mice 2 days after Dox exposure when compared to ZsGreen– Aire+ MHC^hi^ mTECs (Fig. 3D) further underscoring the Aire‐dependency of β5t expression. Given the relatively short half‐life of 7–8 days for Aire‐expressing mTECs 29, these findings suggested that an early detection of labeled mTECs was primarily the result of promiscuous gene expression and that ZsGreen+ mTECs were progressively derived from precursor cells via a MHC^low^ phenotype (Fig. 2A).

### Postnatal β5t^+^ cTECs marked early in Dox‐treated 3xtg^β5t^ mice contribute to mTEC lineage

**Figure 4:**
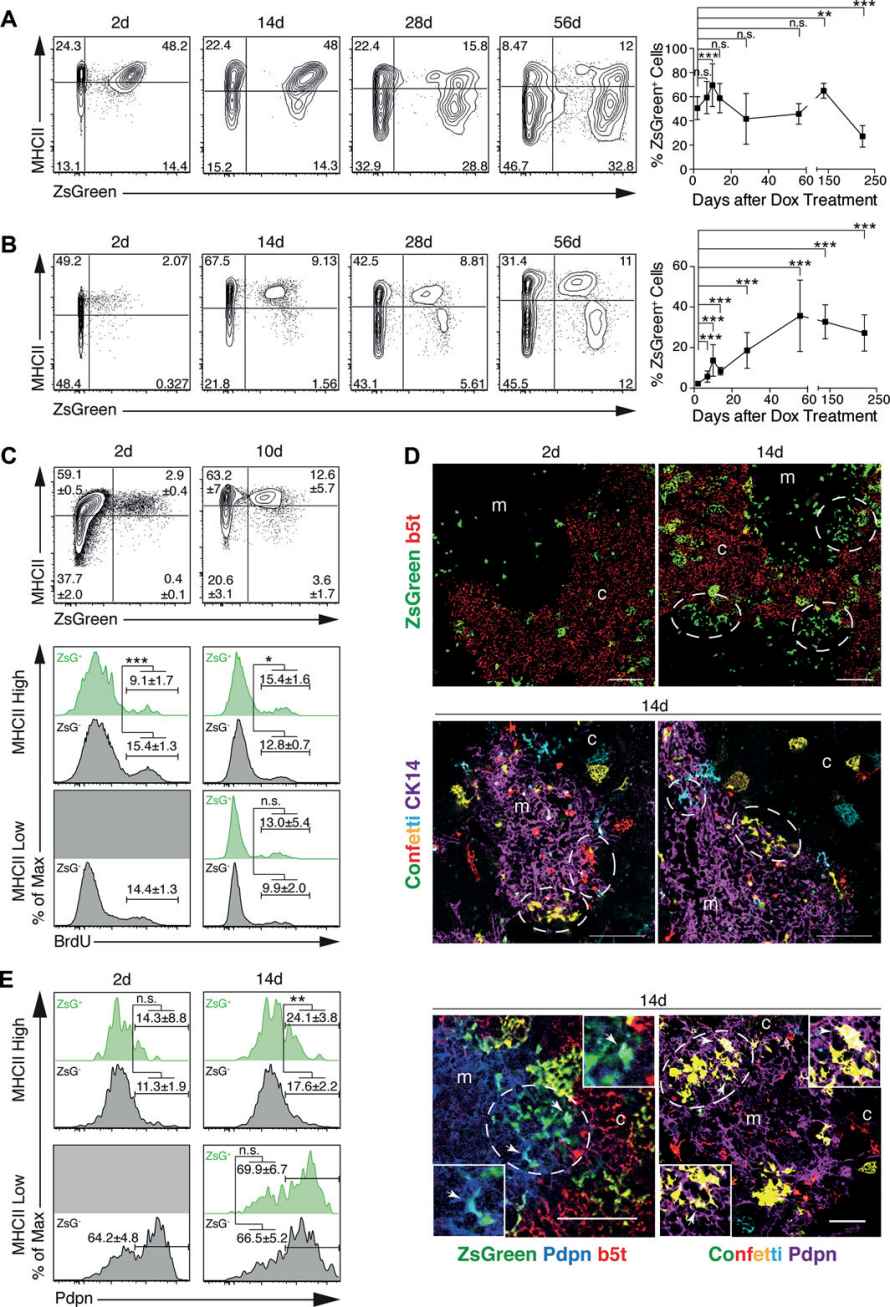
Lineage tracing in 1‐week‐old 3xtg^β5t^ mice. (A) Frequency and time course analyses of ZsGreen expression in cTECs. One‐week‐old mice received a single i.p. injection of Dox (1 mg) and were subsequently analyzed at the indicated times. Flow cytometric analyses of cTECs (EpCAM^+^LY51^+^UEA1^−^CD45^−^) for the expression of ZsGreen and MHCII (left panel) and relative frequencies of ZsGreen+ cTECs over the course of 250 days after treatment (right panel). (B) Frequency and time course analyses of ZsGreen expression in mTECs from mice described in (A). Flow cytometric analyses of mTECs (EpCAM^+^LY51^−^UEA1^+^CD45^−^) for the expression of MHCII and ZsGreen (left panel) and relative frequencies of ZsGreen+ mTECs over the course of 250 days after drug treatment (right panel). (C) BrdU incorporation analysis of mTECs (EpCAM^+^UEA1^+^CD45^−^) in 3xtg^β5t^ mice treated at 1 week of age and chased for 2 and 10 days, respectively (top). The BrdU incorporation rates are displayed in lower panels for ZsGreen– (gray) and ZsGreen+ (green) mTEC subpopulations expressing high or low levels of MHCII. (D) Immunofluorescent analysis of thymic sections from 3xtg^β5t^ mice that had been treated with Dox at 1 week of age and analyzed 2 and 14 days after treatment for the expression of β5t (red) and ZsGreen (green) (top). Note the clusters of ZsGreen positive cells positioned at the cortico‐medullary junction (dashed circles). R26R‐confetti mice were crossed with β5t‐rtTA and LC1 generating triple transgenic mice, 3xtg^confetti^ [β5t‐rtTA::LC1::R26R‐confetti] (bottom). Triple transgenic mice were treated with a single dose of Dox (1 mg) at 1 week of age and analyzed 2 weeks later for the expression of the transgenic fluorochromes and cytokeratin 14 (as mTEC marker). Note monochromatic cell clusters exclusively localized to the cortico‐medullary junction. Images representative from two experiments with a total of three mice each are shown. (E) Flow cytometric analysis of Podoplanin expression in ZsGreen‐negative and ‐positive subpopulations of MHCII^hi^ and MHCII^lo^ mTECs (EpCAM^+^LY51^−^UEA1^+^CD45^−^) (left). One‐week old 3xtg^β5t^ mice were treated with a single dose of Dox (0.3 mg) and analyzed at 2 and 14 days, respectively. Immunofluorescent analysis of thymic sections from 3xtg^β5t^ and 3xtg^confetti^ mice treated at 1 week of age with a single dose of Dox (0.3 mg) and analyzed 14 days later for the expression of Podoplanin (right). Arrows indicate cells coexpressing reporter and Podoplanin in areas that are shown in the close‐up. c: cortex; m: medulla. Scale bar 100 μm. The data signify mean ± SD and are representative of 2–5 independent experiments, each performed with at least three mice per time point. Statistical significance determined by unpaired two‐tailed Student's *t*‐test. *p < 0.05, **p < 0.01, ***p < 0.001.

To test the precursor potential of postnatal β5t^+^ TECs and probe their competence to give rise to the mTEC lineage, we extended our experiments to the analysis of 1‐week‐old 3xtg^β5t^ mice in which the cellularity of the thymic medulla exponentially increases 30. A single Dox injection resulted in these mice in a high and consistent labeling of cTECs (Fig. 4A). Since previous studies had estimated the turn‐over of cTECs and mTECs to be 7–14 days in young mice 30, 31, the recovery of a significant percentage of ZsGreen+ cTECs as late as 240 days after recombination suggested that precursors had been initially labeled that could give rise to mature ZsGreen+ cTECs (Fig. 4A, right panel).

Following Dox treatment of 1‐week‐old 3xtg^β5t^ mice, the frequency of ZsGreen+ mTEC was initially very low and largely restricted to cells with an MHC^hi^ phenotype (Fig. 4B, left panels), including Aire+ and – cells located throughout the medullary compartment (Supporting Information Fig. 4A). However, both the frequency of labeled mTECs as well as that of MHC^lo^ ZsGreen+ mTECs increased progressively after Dox treatment and plateaued at 8 weeks (Fig. 4B, right panel). This kinetic difference in mTEC labeling suggested that β5t‐expressing cells must have served as precursors to the mTEC lineage and that these cells required up to 8 weeks until their progeny established a ratio of MHC^hi^ and MHC^lo^ populations similar to that of nonlabeled mTECs.

Two days after Dox treatment, labeled MHC^hi^ mTECs displayed a lower proliferation rate compared to the population of unlabeled MHC^hi^ mTECs (Fig. 4C), a finding consistent with the notion that β5t+ MHC^hi^ mTECs as other Aire^+^ mTECs represent mostly postmitotic cells 32. Ten days after Dox treatment, MHC^hi^ ZsGreen+ mTECs proliferated at an increased rate implying that this population now comprised a higher frequency of maturing mTECs (Fig. 4C).

We next sought to localize these precursor cells and their immediate clonal progeny within the postnatal thymus. Tissue sections taken 2 weeks after initiation of Dox treatment demonstrated multiple clusters of ZsGreen+ mTECs at the cortico‐medullary junction (Fig. 4D). In contrast, the pattern of labeled mTECs in the other parts of the medulla remained unchanged. After a longer chase, ZsGreen+ mTECs were found more evenly distributed throughout the entire medulla (Supporting Information Fig. 4C). These tracing studies thus indicated an expansion of mTEC precursors but could not inform on their clonality. We therefore generated [β5t‐rtTA::LC1::R26R‐Confetti] mice (designated 3xtg^confetti^) that allow for a monochrome labeling of individual β5t^+^ TECs and their progeny 33. After a single dose of Dox at 1 week of age, thymus tissue was isolated 2 weeks later and sections were screened for fluorochrome‐labeled cell clusters. Small aggregates of 5–25 TEC expressing the same single fluorochrome could be detected at the cortico‐medullary junction of treated but not untreated 3xtg^confetti^ mice (Fig. 4D and data not shown). Because mTEC progenitors located at the cortico‐medullary junction have been demonstrated to express Podoplanin 34, we determined the frequency of Podoplanin‐positive, ZsGreen‐positive, and ‐negative TECs in 3xtg^β5t^ mice treated at one week of age with a single dose of Dox. Though all ZsGreen+ cTECs expressed Podoplanin (Supporting Information Fig. 4B), this marker was detected only on a fraction of ZsGreen+ mTECs (day 2: 14%; and day 14: 25%) (Fig. 4E). The expression of Podoplanin therefore identifies a subpopulation of the newly generated mTECs. In keeping with this finding, Podoplanin‐positive cells were detected within the emerging, ZsGreen‐positive clusters at the cortico‐medullary junction (Fig. 4E). In aggregate, these data strongly suggested that β5t‐expressing precursors localized at the junction between cortex and medulla had proliferated and contributed to the growth of the epithelial component of the thymic medulla.

### Postnatal β5t‐positive cTECs serve as precursors for mTECs

**Figure 5:**
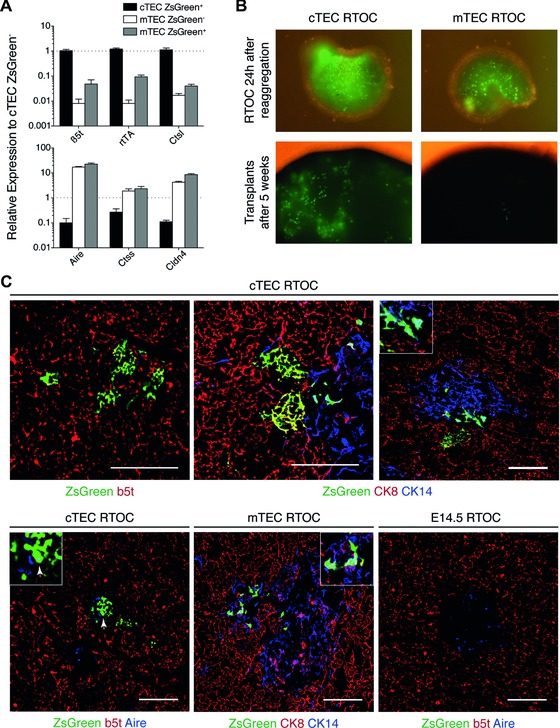
Transplantation of ZsGreen+ cTECs from 3xtg^β5t^ mice gives rise to cortical and medullary TECs. (A) RT‐qPCR gene expression analysis of cortical (β5t, rtTA, Ctsl) and medullary (Aire, Ctss, Cldn4) TEC markers in ZsGreen–/+ TEC subpopulations. One‐week‐old mice received a single i.p. injection of Dox (1 mg) 48 h prior to sorting cTECs (EpCAM^+^LY51^+^UEA1^−^CD45^−^) and mTECs (EpCAM^+^LY51^−^UEA1^+^CD45^−^), subdivided by the expression of ZsGreen. Gene expression was normalized to EpCAM and is presented as relative expression to cTEC ZsGreen– (mean ± SD, *n* = 3). (B) Representative macroscopic images of RTOC 24 h after reaggregation (top) and the transplants 5 weeks posttransplantation (bottom). (C) Immunofluorescent analysis of thymic sections from transplants made with ZsGreen+ cTECs for the expression of ZsGreen and β5t (top left), ZsGreen, CK8 and CK14 (top middle, right) or ZsGreen, β5t and Aire (bottom left), and the analysis for the expression of ZsGreen, CK8, and CK14 in thymic sections from transplants made with ZsGreen+ mTEC (bottom middle) or for ZsGreen, β5t, and Aire in transplants originally using embryonic stromal cells alone (bottom right). Arrows indicate cells coexpressing ZsGreen and mTEC marker (CK14 or Aire) in areas that are shown in the close‐up. Single images representative from of three experiments with a total of two mice each are shown. Scale bar 100 μm.

Data presented so far suggested that precursors to the mTEC lineage express β5t and are resident at the cortico‐medullary junction from where their progeny extends into the medulla early in postnatal life. However, the efficiency with which these precursors seemingly contributed to the mTEC compartment already decreased in the second week of life (Supporting Information Fig. 4C). To test formally whether postnatal β5t+ cTECs served as mTEC precursors, ZsGreen+ cTECs were isolated from 1‐week‐old 3xtg^β5t^ mice 2 days after Dox treatment, reaggregated with wild type, nonhaematopoietic E14.5 thymic stromal cells (1.5 × 10^5^) and grafted as organoids under the kidney capsule of adult athymic (*nu/nu)* recipients. ZsGreen+ mTECs isolated in parallel served as the corresponding controls. A selection of exemplary cortical and medullary TEC genes, respectively, used to characterize further the grafted cells revealed the expected differences in transcription profiles (Fig. 5A). Notably, markers characterizing thymic epithelial progenitor cells 17, 18 were not differentially expressed in ZsGreen+ cTECs relative to ZsGreen‐ cTECs (Supporting Information Fig. 5A) as the former constitute a population of cells at different developmental stages precluding an easy identification of the mTEC precursor potential of β5t+ cTECs.

The microscopic analysis of reaggregate thymic organ cultures (RTOC) demonstrated the presence of ZsGreen+ TECs in both types of grafts as early as 24 h after forming organoids (Fig. 5B, upper panels). Five weeks after the placement of grafts under the kidney capsule (Supporting Information Fig. 5B), the transplanted tissue was further investigated (Fig. 5B, lower panels, and Fig. 5C). The number of ZsGreen+ TECs in either the cortex or the medulla was limited owing to the ratio of labeled to unlabeled stromal cells (1:6) used to generate RTOC and the demonstrated difference in cell proliferation between embryonic and postnatal TECs 31. Tissue sections of grafts in which ZsGreen+ cTECs were admixed with E14.5 wild‐type stromal cells demonstrated not only the presence of ZsGreen+ TECs in the cortex but also disclosed mTEC clusters that expressed the typical medullary markers cytokeratin 14 (CK14) and Aire (Fig. 5C, upper panels). In contrast, tissue sections from RTOC generated with ZsGreen+ mTECs revealed labeled TEC only in the medulla where the cells displayed an identical phenotype to medullary epithelia derived from ZsGreen+ cTECs (Fig. 5C, lower panels). Taken together, these transplantation studies unequivocally demonstrated that within 5 weeks after engraftment ZsGreen+ cTECs gave rise to TECs both in the cortex and the medulla whereas ZsGreen+ mTECs contributed exclusively to the epithelial compartment of the medulla highlighting an essential difference in the developmental potential of these two postnatal cell populations.

## Discussion

TEC patterning is initiated during fetal development and continues throughout postnatal life as reflected by a permanent replacement of TECs in both cortex and medulla 30, 31, 35. The precise developmental point and physical location at which cTECs and mTECs diverge has, however, remained undefined. During embryonic development, the majority of mTECs, including Aire^+^ cells, derive from β5t‐expressing progenitors 15. Using in vivo lineage‐tracing at population and single cell resolution, we now demonstrate that individual β5t^+^ cortical progenitors located at the cortico‐medullary junction contribute to the formation and maintenance of the postnatal medulla. This input parallels the expansion of the thymic medulla and its extent is maximal during the first week of life. Thus, age determines the degree by which β5t^+^ cortical precursors contribute to the medullary epithelial compartment, revealing a gradual change in the precursor‐progeny relationship within the mTEC lineage. These results contribute to an evolving concept that identifies differences between TEC lineage development in the embryo 8, 9, 10, 11 and TEC maintenance in the postnatal thymus 16, thus highlighting a unique spatio‐temporal contribution of β5t^+^ cortical epithelial precursors to the medullary TEC compartment.

Several developmental models have been suggested to explain the step‐wise formation and maintenance of the thymic epithelial scaffold 14. Single epithelial precursors with a developmental potential to contribute to both cortex and medulla can be isolated at E12.5 from the thymus anlage 9, 36 and TECs with either a cortical (CD205, β5t) or medullary phenotype (MTS10, Claudin3/4) are detected at that time 20, 21, 37, 38. The precise phenotype of TEC progenitors and hence the developmental stage at which these cells reduce or even lose their bipotency in lieu of an exclusive contribution to a single TEC lineage remains yet to be defined and may likely differ between fetal and postnatal mice 21.

Distinct maturational stages have also been described for the postnatal mTEC lineage where a single linear differentiation process extends from immature progenitors (MHCII^lo^ CD80^lo^ Aire^−^) to mature epithelia (MHCII^hi^ CD80^hi^ Aire^+^) that may discontinue their Aire expression at a terminal stage 39, 40. Recently SSEA‐1^+^ Claudin3/4^+^ mTECs have been identified that display a remarkable self‐renewing capacity and serve as lifelong progenitors for the medullary but not the cortical epithelial compartment 22. Given their specific developmental potential, SSEA‐1^+^ Claudin3/4^+^ mTEC progenitors must be distinct from the cortical β5t^+^ precursors described here though they may represent a first unipotent progeny downstream of cortical β5t^+^ precursors. Moreover, our kinetic studies would suggest that cortical β5t^+^ precursors represent a rare population because following Dox treatment of 1‐week‐old 3xtg^β5t^ mice up to 56 days are required to achieve maximum labeling of the mTEC compartment. Assuming that mature mTECs are still largely derived from β5t^+^ precursors under these conditions (comparable to the fetal labeling experiments), we reason that their frequency must be low as a significant expansion is needed to label eventually as many as one third of all mTECs after a single Dox dose. This interpretation is in keeping with our finding that single clonal TEC clusters are detected at the cortico‐medullary junction of individual medullary islets within 2 weeks following Dox treatment and that this anatomical location constitutes the site of a recently detected, Podoplanin‐positive mTEC precursor 34. Indeed, a small fraction of Podoplanin‐positive cells was detected among labeled mTECs shortly after treatment and the frequency of Podoplanin‐positive cells increased among ZsGreen+ mTECs 2 weeks after Dox treatment. But whether other cTEC‐associated markers are expressed within this newly generated, immature mTEC population, such as the recently reported chemokine (C–C motif) receptor‐like 1 (CCRL1) 41, remains to be tested. The reconstitution efficiency of early‐labeled β5t^+^ cTECs was however low, which is, at least in part, due to the competitive growth advantage of fetal over adult TECs used in the transplantation experiments.

In view of the labeling kinetics observed, it is likely that in young mice cortical β5t^+^ precursors with a developmental potential for the mTEC lineage give first rise to a larger number of epithelia that lack β5t expression but have a self‐renewing capacity and can replenish mTECs at all ages to suit the homeostatic needs of the medulla. This population of epithelia may represent the earliest post β5t‐stage in the mTEC lineage and could function comparable to transit‐amplifying cells (TAC) that balance precursor usage with tissue generation 42. Once the exponential growth of the medulla—and its possible physical stress on the surrounding tissue—has seized, mTEC development may largely be drawn from the TAC‐like population with cortical β5t^+^ precursors contributing now only to a limited extent to the maintenance of the medulla. Although our data strongly favor this mechanism of medulla formation and maintenance, we cannot exclude that a smaller fraction of β5t+ progenitor cells was labeled in adult mice when compared to mice at 1 week of age.

Our results together with previously published data 19 propose in aggregate that individual islets are generated during fetal organogenesis from a single precursor and later in life are of oligoclonal origin. While it had so far remained untested whether these epithelial progenitors are only able to establish the correct medullary architecture during ontogeny, lineage‐tracing, and competitive grafting experiments presented here now demonstrate that this capacity of β5t^+^ cortical epithelial precursors is maintained into postnatal life. However, contributions from these precursors are significantly diminished in older mice and are not reactivated following thymic injury in adult mice (data not shown) thus inferring a regenerative process that is compartment‐intrinsic.

The persistence of ZsGreen^+^ TECs in both cortex and medulla over an extended period following short‐time labeling is remarkable and can best be explained by a pool of long‐lived precursors in which recombination had successfully occurred and from which mature TECs are eventually generated, though alternative mechanisms may exist. For example, a fraction of differentiated ZsGreen+ TECs could be generated in situ via a process of self‐duplication in the absence of any contribution from progeny of labeled precursor cells, a phenomenon already reported for other epithelial cell lineages 26, 27, 28. However, this mechanism would need to be stochastic and independent of specific developmental niches, as individual clusters of ZsGreen^+^ TEC cannot be discerned in the cortex or the medulla of older mice that had been treated with Dox at 1 or 5 weeks of age.

This work extends recently published data 43 providing important insights regarding the anatomical location and clonal progeny of β5t^+^ precursor derived mTECs. Moreover, grafting experiments provide direct evidence that postnatal β5t^+^ cTECs contain precursors for mTECs.

In summary, we demonstrate to our knowledge for the first time that a population of β5t^+^ cortical progenitors positioned adjacent to the medulla gives rise to mTECs. The extent of this contribution changes considerably during the first postnatal weeks when number and proportion of mTECs dramatically increase due to extensive proliferation. The precise signals and their downstream molecular events responsible for this change remain presently undefined. However, insight into this process and the isolation and manipulation of β5t^+^ cortical epithelial precursors constitute a novel rationale for therapeutic strategies to restore immune function.

## Materials and methods

### Mice

C57BL/6 mice were obtained from Janvier (France). LC1‐Cre transgenic, CAG‐loxP‐STOP‐loxP‐ZsGreen, CAG‐loxP‐stop‐loxP‐EGFP, R26R‐Confetti, β5t‐Cre, and tetO‐Cre1Jaw/J mice were described previously 15, 24, 25, 33, 44, 45. β5t‐rtTA mice were generated analogous to the β5t‐Cre animals previously reported 15. The day of a visible vaginal plug was designated in timed pregnancies as embryonic day 0.5 (E0.5). Mice were kept under specific pathogen‐free conditions. Experiments were in accordance with local and national regulations and permissions.

### Doxycycline treatment

Fetal [β5t‐rtTA::LC1‐Cre::CAG‐loxP‐STOP‐loxP‐ZsGreen] mice (designated triple transgenic, 3xtg^β5t^) were exposed from E7.5 until birth to Doxycycline (Dox) via the mother's drinking water (Sigma, 2 mg/mL in sucrose (5% w/v)). One‐week‐old 3xtg^β5t^ mice were treated with a single i.p. injection of Dox (0.3 mg) whereas older mice received two i.p. injections of Dox (2mg, each) in the course of 24 h during which they were also exposed to drinking water supplemented with the drug.

### BrdU labeling

Mice were injected i.p. with 1mg BrdU (BrdU Kit, BD Pharmingen) and kept for 16 h prior to analysis.

### Flow cytometry

Cells were prepared as reported elsewhere incubated with antibodies specific for CD45 (30F11; eBioscience), EpCAM (G8.8; DSHB, University of Iowa), MHCII (M5/114.15.2; BioLegend), Ly51 (6C3; BioLegend), UEA‐1 (Reactolab), Pdpn (8.1.1; BioLegend), Aire (5H12; eBioscience), and BrdU. For intracellular staining, cells were fixed, permeabilized (Cytofix/Cytoperm Kit, BD Biosciences), and labeled for the expression of Aire or the incorporation of BrdU. Stained samples were acquired on a FACSAria II flow cytometer and the data were analyzed using the FlowJo (Treestar) software.

### Quantitative PCR analysis

Primer sequences are available upon request. *Epcam*‐specific transcripts were used as an internal control and PCR data were analyzed using the LinRegPCR software 46.

### Transcriptomic analyses

The transcriptomes of 174 single mature mTECs expressing more than 3000 genes (GEO accession GSE60297; 7 were examined for Psmb11 expression. 26 cTEC and 229 mTEC marker genes (Fig. 3C) were identified from cTEC and Aire‐KO mTEC population RNA‐seq data (GEO accession GSE53110). Marker genes were required to be expressed at > 20 FPKM in the population of interest and < 1 FPKM in the other. Aire‐regulated genes and TRA definitions are taken from 7.

### Histological analyses

Fixed in formalin and dehydrated overnight, thymic tissues were frozen, sectioned, and stained for Psmb11 (MBL), CK8 (Progen), CK14 (Covance), Pdpn (BioLegend), and Aire (eBioscience). Images were acquired using a Zeiss LSM510 (Carl Zeiss).

### RTOC transplants

Twenty‐five thousand sorted transgenic TECs were mixed with 150,000 wild‐type embryonic (E14.5) cells depleted of CD45^+^ and Ter119^+^ subpopulations, spun down and incubated over night at 37°C. Reaggregates were placed under the kidney capsule of the recipient *nu/nu* mice and analyzed 5 weeks later.

### Statistical analyses

Statistical analyses were performed using Students *t* test (unpaired, two‐tailed). Probability values were classified into four categories: *p* > 0.05 (n.s.), 0.05 ≥ *p* > 0.01 (*), 0.01 ≥ *p* > 0.001 (**), and *p* ≤ 0.001 (***).

## Conflict of Interest

The authors declare no financial or commercial conflict of interest.

**Abbreviations**
Dox: doxycycline
RTOC: reaggregate thymic organ culture
rtTA: reverse tetracycline transactivator
TAC: transit‐amplifying cell
TEC: thymic epithelial cell
TRA: tissue restricted antigen

## Supporting information

As a service to our authors and readers, this journal provides supporting information supplied by the authors. Such materials are peer reviewed and may be re‐organized for online delivery, but are not copy‐edited or typeset. Technical support issues arising from supporting information (other than missing files) should be addressed to the authors.

Supporting information
