# Supplementary material for: Dynamic spatio‐temporal contribution of single β5t+ cortical epithelial precursors to the thymus medulla
Source: Eur J Immunol. 2016 Jan 18;46(4):846–56. doi: 10.1002/eji.201545995 (PMC4832341; doi:10.1002/eji.201545995)
Supplement: Supplementary file 1 — Supporting information [file EJI-46-846-s001.pdf]

Peer review correspondence

European Journal of Immunology

Dynamic spatio-temporal contribution of single β5t^{+} cortical epithelial precursors to the thymus medulla

Carlos E. Mayer, Saulius Žuklys, Saule Zhanybekova, Izumi Ohigashi, Hong-Ying Teh, Stephen N. Sansom, Noriko Shikama-Dorn, Katrin Hafen, Iain C. Macaulay, Mary E. Deadman, Chris P. Ponting, Yousuke Takahama and Georg A. Holländer

Corresponding author: Georg A. Holländer, Department of Biomedicine, University of Basel, Basel, Switzerland

---

Review Timeline:	Submission date:	10 August 2015
	First editorial decision:	9 September 2015
	Revision received:	24 November 2015
	Accepted:	17 December 2015

Handling Executive Committee member: Prof. Bernard Malissen

Please note that the correspondence below does not include the standard editorial instructions regarding preparation and submission of revised manuscripts, only the scientific revisions requested and addressed.

First Editorial Decision – 9 September 2015

Dear Prof. Holländer,

Manuscript ID eji.201545995 entitled "Spatio-temporal contribution of single β5t+ cortical epithelial precursors to the thymus medulla", which you submitted to the European Journal of Immunology, has been reviewed.

The comments of the referees are included at the bottom of this letter. Although the referees have recommended publication, some revisions to your manuscript have been requested. Therefore, I invite you to respond to the comments of the referees and revise your manuscript accordingly.

You should also pay close attention to the editorial comments included below. **In particular, please edit your figure legends to follow Journal standards as outlined in the editorial comments. Failure to do this will result in delays in the re-review process.**

If the revision of the paper is expected to take more than three months, please inform the editorial office. Revisions taking longer than six months may be assessed by new referee(s) to ensure the relevance and timeliness of the data.

Once again, thank you for submitting your manuscript to European Journal of Immunology. We look forward to receiving your revision.

Yours sincerely,
Laura Soto Vazquez

on behalf of Prof. Bernard Malissen

Editorial Office
European Journal of Immunology
e-mail: ejied@wiley.com
www.eji-journal.eu

****

Reviewer: 1

Comments to the Author
Mayer et al have used a lineage tracing to study beta5t+ progenitor cells in thymus, and their potential as precursor cells of mTECs. The study is an extension of earlier report on beta5t+ thymic progenitor cells (Ohigashi et al 2014) and addresses a hotly debated contribution of different TEC precursor cells to cTEC and mTEC lineage. The authors show an efficiency of postnatal beta5t+ cells to differentiate into mTEC lineage, and that this potency is decreased at adult age. The paper is well written with valid experimental design and conclusions. A comment is related to the overlap with several other previously published markers of TEC precursors, for example, CD205 and podoplanin. Did the authors attempt to perform immunofluorescence co-stainings or flow analysis of beta5t+ cells with CD205 and/or podoplanin?

Reviewer: 2

Comments to the Author
The manuscript by Mayer et al is a concise, properly designed and well-written study that provides further details regarding our current understanding of events that control adult thymus medulla differentiation and homeostasis. Using a novel genetic inducible fate mapping approach in mouse, the authors show that

European Journal of Immunology

TEC progenitors expressing the cortical marker beta5t+ contribute the cortical and medullary lineage in the postnatal thymus. These results provide evidence that bipotent TEC progenitors nestle within the cortex of the adult thymus.

The authors engineered transgenic mice that express the reverse tetracycline transactivator (rtTA) under the control of Psmb11 (beta5t) locus, and crossed them with LC1 mice, in which Luciferase and Cre recombinase are expressed under the control of a rtTA responsive promoter, and ZsGreen reporter animals, in which ZsGreen reporter gene is ubiquitously expressed under the control of CAG promoter once the loxP-flanked stop sequences is excised by Cre expression. In these triple transgenic mice, ZsGreen expression purportedly indicates current and/or past expression of beta5t in cells during/following doxycycline (Dox) treatment.

The authors started by performing labelling experiments during embryonic life and, in line with their previous observations, confirmed that around 80% of cTEC and mTEC in the young adult thymus derived from beta5t+ fetal precursors (Figure 1). Similar findings were obtained when Dox treatment was performed at 1 week of age (Figure 3), albeit one can notice already a drop in the deepness of labelling. As the half-life of cTEC and mTEC is estimated to be around 1-2 weeks, these findings already infer, as also discussed by the authors (page 13), that labelled cTEC and mTEC at 4-7 weeks post-treatment are likely maintained/renewed through bipotent and/or compartment-restricted progeny that exist downstream of beta5t progenitors, and are presumable established during early postnatal life, a period in which the thymic epithelial cells are actively expanding (Gray Blood 2006, Dumont-Lagace J Immunol 2014, Ribeiro EJI 2014).

1. The labelling experiments in the adult thymus are of a more complex interpretation. The conclusion that beta5t progenitors have a limited contribution to the medulla in the adult thymus (e.g. abstract lines 16/17, page 4 lines 23/24, page 11 lines 26/28, page 14 lines 14), in contrast to the fetal and postnatal counterparts, raises one of my major concerns in this study. It is important to note that the labelling efficiency of beta5t within adult cTEC is of equal limited penetrance (supplementary figure 2A). Moreover, there is some discrepancy between the detection of beta5t protein and ZsGreen+cTECs (supplementary figure 2B), which leads to the question if labelling within beta5t-expressing cTEC is effectively achieved. Can the authors comment on this? Does this discrepancy reflect alterations (spikes of activity) in the beta5t promoter within cTECs and/or half-life of beta5t protein? Can the authors exclude the possibility that non-labelled beta5t protein+ cTEC are equally contributing to the mTEC network, albeit one can't measure that? I reckon that in this scenario, one cannot ascertain that beta5t progenitors have a limited contribution to the medulla in the adult thymus. The authors should comment on this possibility.

2. The authors referred that prolonged Dox-treatment did not increase the labelling efficiency and that thymic injury in the adult mice did not reactivate beta5t precursors (page 14). Can the authors provide

details of the thymic injury experiment? Was it induced by radiation or glucocorticoid treatment, and were mTEC fully depleted in that scenario? One can reason that the incomplete depletion of mTEC (and their precursors) might preclude reactivating a beta5t-labelle progeny? Was there any enhanced labelling detected in cTEC following recovery?

3. Also related with point 1, did the authors compare/measure the complementary luciferase activity in the postnatal and adult thymus in the different labelling experiments?

4. The authors reported interesting clusters of ZsGreen+ at the cortico-medullary that arise 14 days post-Dox-treatment of 1 week-old mice, and showed that these clusters are clonal by employing an elegant triple transgenic beta5t-rtTA:LC1:R26R-Confetti mice. They conclude that while clonal during embryonic life, the medullary islets of the adult thymus might be oligoclonal, presumably as a result of the contribution of b5t precursors emanating from the cortex (page 13 line 43). Was the dynamic of these clusters analysed beyond day 14? Do they spatially engraft in the central areas of the medulla network? And do they express gp38 (Onder EJI2015)?

5. The notion that developmental stage-specific pathways participate in the organization of the adult mTEC niche is temporally in line with a previous study that identify a novel subtypes of CD80-Aire- and CD80+Aire+mTEC arising during the first week of postnatal life and expressing intermediate levels of CCRL1, an atypical chemokine receptor highly expressed in cTEC (Ribeiro EJI 2014). It would be of potential interest to refer to this study in the discussion (page 13 lines 9-37) as further support this notion.

6. Given that few mTEChi express beta5t as a result of Aire-driven promiscuous gene expression (Figure 3), can the authors exclude the possibility that (at least part of) these clusters derived from the expansion of Aire+mTEChi cells already existing on day 2 post-labelling (Figure 4B)? Although ZsGreen+(Aire+?)mTEChi cells appear to proliferate less than ZsGreen neg mTEChi counterparts on day2, they still expand (9%) and catch up on day14. Do ZsGreen+ cells found within the medulla and borders of b5t-protein+ cells (Figure 4D 2d) express Aire protein? And can this alternative scenario be considered?

7. Along these lines, it is interesting to note a difference in the level of ZsGreen in mTEChi and mTEClo cells. mTEChi and mTEClo cells appear to express lower and higher levels of ZsGreen, respectively (Figure 4B, mostly clear at days 28 and 56). Do this difference in the labelling intensity of ZsGreen reflect alterations in the activity of the CAG promoter between mTEChi and mTEC lo? Or could this indicate distinct origins/waves of ZsGreen+ mTEC?

8. In the last part of the study, the authors use the RTOC system (Figure 5) and provide compelling evidence that ZsGreen+ cTEC engraft within the thymic environment and are found both in the cortex and

European Journal of Immunology

medullary network. Do ZsGreen+ cells found within the cortex express other cTEC markers (e.g. beta5t protein, Ly51, Cd205) apart of CK8+ (Figure 5C)? The authors only show beta5 protein staining in medullary ZsGreen+ cells.

Other points

9. Introduction (page 3 line 31). Apart of negative selection, mTEC are equally important for regulatory T cell differentiation (Cowan JEM 2013). It would be of potential interest to recognize this evidence.

First revision – authors' response – 24 November 2015

Reviewer #1

We thank the reviewer for his/her helpful comments and suggestions. In answering the points made, we believe that the manuscript has improved in clarity and now provides additional information of relevance.

"A comment is related to the overlap with several other previously published markers of TEC precursors, for example, CD205 and podoplanin. Did the authors attempt to perform immunofluorescence co-stainings or flow analysis of beta5t+ cells with CD205 and/or podoplanin?"

We appreciate the suggestion made and therefore analyzed 3xtgβ5t and control mice for the expression of CD205 and podoplanin by ZsGreen+ TEC. One week old mice were treated with a single dose of Doxycycline (0.3mg). Two and 14 days later, TEC were isolated and analysed by flow cytometry for the expression of ZsGreen, CD205 and Podoplanin. At each of these time points, ZsGreen-positive and ZsGreen-negative cells expressed comparable frequencies of CD205 (data not shown). All ZsGreen+ cTEC expressed Podoplanin (see revised Supporting Figure 4 B), whereas only a fraction of ZsGreen+ mTEC expressed Podoplanin (day2: 14%, and day 14: 25%). Podoplanin-positive ZsGreen+ mTEC after 14 days of "chase" may therefore likely include newly generated mTEC. In keeping with this interpretation, Podoplanin-positive cells were detected within the ZsGreen-positive clusters emerging at the cortico-medullary junction in "chase" experiments. This result is now included in the Results and displayed in panel E of the revised Figure 4.

Reviewer #2

European Journal of Immunology

We welcome the reviewer's comments and suggestions related to our "elegant studies" that provide "compelling evidence". These notes have helped to significantly improve our manuscript. In response, we provide below a point-by-point response and indicate the changes made in the revised manuscript:

The reviewer raises the question whether "labelling within β5t-expressing cTEC is effectively achieved" and whether this "reflects alterations (spikes of activity) in the β5t promoter within cTECs and/or half-life of β5t protein?"

To address this point, we determined the expression of rtTA in cTEC and mTEC isolated from heterozygous 3xtgβ5t mice at 1 and 5 weeks of age. We found that the rtTA expression levels were significantly reduced in 5 week old mice in both cTEC and mTEC (this data is included in Supporting Figure 3, panel A). This age-related difference in rtTA expression (under the transcriptional control of the Psmb11 locus) may likely account, at least in part, for the reduced recombination efficiency observed in TEC of mice 5 weeks of age and older.

The reviewer queries whether β5t progenitors have a limited contribution to the thymic medulla of an adult mouse and asks to comment on this possibility.

Relevant changes in the frequency of ZsGreen+ mTEC were not observed in 3xtgβ5t mice treated with Doxycycline at 1 week of age and followed for at least 20 weeks (Figure 4 B). This phenotypic stability strongly argues for an initial contribution of ZsGreen+ epithelial precursors to the medullary compartment that is not further "diluted" with time by non-labeled β5t+ precursors that have newly been generated after exposure to Doxycycline. We therefore conclude that contributions of un-labeled β5t+ precursors to the adult medulla can sensibly be excluded as an explanation for our findings. However and as pointed out by the reviewer, the possibility remains that a smaller fraction of b5t+ progenitor cells was labeled in adult mice when compared to mice at one week of age. this point is now mentioned in the revised manuscript (page 14).

The reviewer requests further details how the thymus was injured and queries whether an "incomplete depletion of mTEC (and their precursors) might preclude reactivating a β5t-labelled progeny".

Sub-lethal irradiation (550 rad) was used to cause thymic injury in 6 week-old 3xtgβ5t mice treated one week earlier with a single dose of Doxycycline (2mg) and 24 hours of water supplemented with the drug (2mg/ml). Two days after irradiation, total thymus cellularity had dropped by 96% in comparison non-irradiated controls (3±0 x 106 vs. 114±8 x 106 per thymic lobe). Concurrently, mTEC cellularity decreased from 230'000±23'000 in non-irradiated 3xtgβ5t mice to 81'000±10'000 cells, demonstrating a significant reduction in response to injury. It is however of note, that even under conditions of lethal irradiation the population of TEC is not entirely ablated as these cells are, in contrast to thymocytes, fairly radio-resistant.

The reviewer asks whether there was "any enhanced labelling detected in cTEC following recovery".

The percentage of ZsGreen+ cTEC and mTEC was measured at 2 days and 7-10 weeks after sub-lethal total body irradiation. The frequency of labeled cTEC, and mTEC did not significantly change at any of the two time points (data not shown), suggesting that ZsGreen+ TEC did not preferentially reconstitute following thymus irradiation.

The reviewer asks whether we did "compare/measure the complementary luciferase activity in the postnatal and adult thymus in the different labeling experiments"

The experiments deliberately used the expression of Cre to lineage trace and quantify the progeny of TEC that have (at one point in their differentiation) expressed the Psmb11 locus. Luciferase activity was therefore not further investigated.

The reviewer asks whether the cell clusters detected at the cortico-medullary junction of 3xtgβ5t mice treated at one week of age with Doxycyline express gp38, persist beyond day 14 and engraft in the central areas of the medullary network.

All ZsGreen+ cTEC stained positively for Podoplanin (see revised Supporting Figure 4, panel B), whereas only a fraction of ZsGreen+ mTEC was positive for this marker (day 2: 14% at 2d, and day 14: 25%; see Figure 4, panel E). Podoplanin-positive, ZsGreen+TEC were also detected in the cell clusters at the cortico-medullary junction. These results demonstrate that a fraction of newly generated mTEC and their progeny expresses Podoplanin. Whether this cell surface molecule exclusively identifies early mTEC precursors can not be inferred from the experimental conditions employed here.

Two weeks after a single dose of Doxycycline (0.3mg), ZsGreen-labeled cell clusters were almost exclusively observed at the cortico-medullary junction of 3xtgβ5t and 3xtg R26R-Confetti mice (Figure 4 D). However, these cells were scattered throughout the medulla at later time points (see revised Supporting Figure 4, panel C).

The reviewer suggests to mention in a revised Discussion the study of Ribeiro and colleagues (EJI, 2014) identifying "novel subtypes of CD80-Aire- and CD80+Aire+mTEC arising during the first week of postnatal life and expressing intermediate levels of CCRL1, an atypical chemokine receptor highly expressed in cTEC".

We appreciate the reviewer's suggestion and have now made revised Discussion of our manuscript accordingly (see page 13).

The reviewer wishes to know whether labeled cell clusters at the cortico-medullary junction may have derived from mTEChi cells that express β5t as a result of promiscuous gene expression.

Two days after Doxycycline treatment of 1-week-old 3xtgβ5t mice, ZsGreen+ mTEC were detected throughout the medulla with fraction of these cells expressing Aire protein (see new Supporting Figure 4, panel A). Two weeks after treatment, clusters of labeled cells were only detected adjacent to the cortico-medullary junction. This result is in keeping with the previously reported observation that MHCIIhi mTEC are largely post-mitotic cells with a short half-life (Gray et al. J. Exp. Med. 2007; 204:2521-2528) and that the frequency of Aire-positive mTEC did not increase following their labeling and a 2 week chase (Metzger et al. Cell Rep. 2013; 5:166-179). We therefore view it as highly unlikely that Aire+ ZsGreen-labeled MHCIIhi mTEC cells act as “precursors” for ZsGreen-labeled mTEC .

The reviewer notes that “mTEChi and mTEClo cells appear to express lower and higher levels of ZsGreen, respectively, and thus queries whether this difference reflects alterations in the activity of the CAG promoter, or, alternatively, is explained “by distinct origins/waves of ZsGreen+ mTEC.”

Differences in the levels of ZsGreen expression were only observed for mTEC and a reduced expression appeared to be age-dependent only for mTEC MHCIIhi (see Figure 2 A and 4 B). We therefore reason that differences in ZsGreen expression are likely explained by different biology for the individual TEC lineages and seem to be determined by the proliferative history, half-life, and CAG promoter activity in individual TEC subpopulations as well as the protein’s half-life in that respective cell. Our experiments do, however, not provide formal proof for any of these possible explanations.

The reviewer asks whether ZsGreen+ cells in the cortex express cTEC markers other thank CK8 (e.g. beta5t protein)?

During the revision of our manuscript we noted an inaccuracy in legend to Figure 5, panel C. Cells stained in red in this figure express β5t and not CK8, as erroneously indicated in the original submission. We have now corrected this mistake and provide additional immunohistological data depicting ZsGreen+ cTEC expressiing CK8 (revised Figure 5 C, upper row, middle panel).

The reviewer points out that mTEC also serve a role in the differentiation of regulatory T cell (Cowan JEM 2013) and suggests that we mention this fact in the revised manuscript.

We are thankful for the reviewer’s comment and have made the necessary edits to the revised manuscript (see page 3).

European Journal of Immunology

Second Editorial Decision – 11 December 2015

Dear Prof. Holländer,

It is a pleasure to provisionally accept your manuscript entitled "Spatio-temporal contribution of single β5t+ cortical epithelial precursors to the thymus medulla" for publication in the European Journal of Immunology. For final acceptance, please follow the instructions below and return the requested items as soon as possible as we cannot process your manuscript further until all items listed below are dealt with.

Please note that EJI articles are now published online a few days after final acceptance (see Accepted Articles: http://onlinelibrary.wiley.com/journal/10.1002/(ISSN)1521-4141/accepted). The files used for the Accepted Articles are the final files and information supplied by you in Manuscript Central. You should therefore check that all the information (including author names) is correct as changes will NOT be permitted until the proofs stage.

We look forward to hearing from you and thank you for submitting your manuscript to the European Journal of Immunology.

Yours sincerely,
Karen Chu

on behalf of Prof. Bernard Malissen

Dr. Karen Chu
Editorial Office
European Journal of Immunology
e-mail: ejied@wiley.com
www.eji-journal.eu
